# Considering Work Arrangement as an “Exposure” in Occupational Health Research and Practice

**DOI:** 10.3389/fpubh.2020.00363

**Published:** 2020-08-04

**Authors:** Allyson O'Connor, Trevor Peckham, Noah Seixas

**Affiliations:** ^1^Department of Health Services, University of Washington, Seattle, WA, United States; ^2^Department of Environmental and Occupational Health Sciences, University of Washington, Seattle, WA, United States

**Keywords:** non-standard employment, occupational health, work arrangement, temporary work, exposure assessment

## Abstract

Most occupational health research is conducted with the so-called “standard employment relationship” in mind, which entails ongoing, full-time employment for a single employer. Yet mounting evidence suggests the way work is organized is increasingly deviating from this standard model, and that work arrangements themselves—the terms and conditions of employment such as contract type and the extent of directive control over tasks—are important determinants of worker health and safety. However, a lack of clear conceptual definitions or taxonomic system defining the wide variety of economic work arrangements in the contemporary workplace hampers rigorous investigation of their relationship to health. The various forms of “non-standard” employment arrangements—also called non-traditional, alternative, flexible, fissured, precarious, contingent, temporary, atypical, or gig work—may have overlapping attributes, yet ambiguity regarding the character of these arrangements obscures mechanisms that lead to increased health and safety risks. Here, we attempt to clarify work arrangements as a workplace exposure, deserving of specific attention within occupational health and safety research, practice, and policy. We argue that, at minimum, three key features of work arrangements need to be considered: (1) whether an arrangement is permanent or temporary; (2) whether a worker is a contractor or an employee; and (3) whether an arrangement involves more than one firm. We further propose mechanisms linking work arrangements to increased work-related health risks to better inform strategies aimed at protecting the growing non-standard workforce.

## Introduction

Occupational health and safety (OH&S) research, practice, and policy has typically been conducted with the so-called “standard employment relationship” in mind; that is, work of permanent, ongoing duration, with full-time hours, and under a single employer that both directs tasks and has clear responsibilities and obligations to the worker. Yet, work arrangements, defined as employment or contractual relationships between buyers and sellers of labor, are increasingly deviating from this standard conception of employment in contemporary labor markets. “Non-standard” work arrangements—a broad moniker used to describe any of the dozens of work arrangements that differ from the standard employment archetype—represent a growing segment of the workforce and are estimated to account for between 8 and 40% of jobs in industrialized economies (1, 2). With this trend, the health disparities between workers in standard employment relationships and those working in non-standard arrangements are becoming increasingly evident. Non-standard work, variously defined, has been associated with a number of adverse worker health outcomes, including poor self-reported health (3–5), increased injury risk and injury severity (6–9), musculoskeletal complaints (10), and poor mental health (11–13). Consequently, non-standard work arrangements are gaining attention within public and occupational health (1, 14–17).

A key challenge in understanding risks and developing adequate OH&S protections for workers engaged in non-standard work is our relatively underdeveloped understanding of the dynamics between work arrangements and health. To date, standard employment relationships provided OH&S research, practice, and policy a useful organizational model for quantifying workplace hazards and setting and enforcing regulatory standards. This model serves us well, but only for the segment of the working population in these standard organizational contexts. As the labor market continues to fissure in favor of maximizing efficiency and flexibility by employing temporary workers, contractors, or workers hired *ad-hoc* through staffing agencies, conventional approaches to research, interventions, and policy are increasingly inadequate to understand the risks associated with work and to protect workers in non-standard arrangements (18).

The lack of a shared vocabulary or taxonomic system to clearly define work arrangements also hinders our ability to address non-standard work (1, 16). Numerous terms are commonly used interchangeably or imprecisely to describe non-standard work arrangements, such as non-traditional, alternative, precarious, contingent, temporary, gig, or atypical arrangements, among others. However, these labels often fail to contend with the significant heterogeneity of work within modern labor markets, relying on vague distinctions between “standard employment” and everything else.

Consider the important differences that can exist among work arrangements commonly grouped together as “temp work.” Some temp workers are hired directly, for a fixed duration, by an employer who directs their work. Other temp workers may be employed by temporary staffing agencies and move from worksite to worksite where a staffing agency's clients control the workers' day-to-day tasks and working conditions. From a practical perspective, these distinct forms of temp work differ in terms of who directs workers' job tasks and working conditions—i.e., their employer, or their employer's client—which may have implications for worker health and safety through a variety of mechanisms. From an analytical perspective, lumping these distinct arrangements together into a single category of “temp worker” could lead to significant measurement error (19). This lack of clarity, even when work arrangements seem similar in name, hampers rigorous investigation into how work arrangements influence health and obscures the mechanisms that lead to increased health and safety risks.

To address these issues, we propose that work arrangements themselves be considered occupational exposures deserving of explicit attention from OH&S professionals alongside the physical, chemical, and psychosocial workplace hazards already familiar to the field. Our first objective is to describe important components of work arrangements that are useful for identifying and distinguishing between various forms of work. Our second objective is to document potential mechanisms by which non-standard work arrangements may contribute to differential health and safety risk. We focus on the character of arrangements within the formal economy, but many of the proposed mechanisms may also apply to workers in informal arrangements, such as day laborers. Overall, we believe that marshaling the principles and expertise of occupational exposure assessment and epidemiology is uniquely helpful for explaining the health implications of current labor market trends, as well as informing potential interventions to protect workers engaged in the wide variety of existing work arrangements.

## Key Distinguishing Features of Work Arrangements

The character and extent of an organization's directive control over work assignments (i.e., what tasks are to be completed, in what order, and in what period of time) has been consistently recognized in labor studies as the most fundamental distinguishing feature between economic relationships (1, 20, 21), and directive control is paramount to workers' experiences in their jobs. In the management field, Cappelli and Keller (19) compellingly argue that the concept of directive control is a key determinant of work arrangement classification. They highlight two key distinctions: (1) whether directive control is shared between more than one entity; and (2) whether the arrangement is governed by employment law or contract law. From an OH&S perspective, the expected duration of a work arrangement is also critical to workers' experiences in their jobs. Indeed, there is a substantial literature documenting health disparities between those in permanent vs. temporary work arrangements (22, 23)—although, as noted above, these studies have not always distinguished the different forms of temporary arrangements well.

Based on these observations, we suggest that three key dimensions of work arrangements be considered simultaneously to distinguish between forms of work: (1) whether an arrangement is permanent or temporary; (2) whether a worker is a contractor or an employee; and (3) whether an arrangement involves more than one firm. While these are not the only components of work arrangements that have health implications, these characteristics represent three prominent ways in which work arrangements are associated with health and safety risk.

### Permanent vs. Temporary

Permanent (or open) contracts confer an expectation of ongoing paid employment and are a key feature of the standard employment relationship. Conversely, temporary (or closed, non-permanent, or fixed-term) contracts of short-term duration are characteristic of many forms of non-standard arrangements. Temporary work includes direct-hire temp workers, on-call, staffing agency workers, independent contractors, subcontractors, and is also the defining feature the broad category of “contingent employment” (24). Where permanent contracts promote security and familiarity with work tasks and workplaces for workers, temporary work evokes the opposite; and the chronic stress of job insecurity associated with temporary arrangements can lead to adverse mental and physical health (25).

### Employee vs. Contractor

Workers providing paid labor can be employees or contractors. In an employer-employee relationship, an employer has significant directive control over an employee's work. This type of relationship is governed by employment law, meaning that an employer has specific legal responsibilities, including administrative requirements (e.g., paying various taxes and providing benefits) and providing certain labor protections (e.g., responsibility for helping workers manage work-related injuries and illness, minimum wage). Alternatively, within a contracted relationship the purchaser of labor (i.e., the client) has limited or no directive control over the work completed by a contractor (i.e., the worker). The terms of contracted arrangements are specified in advance and enforced by the courts within contract law (19). Further, the client is not responsible for administrative requirements or labor protections provided to the worker under employment law. Consequentially, the misclassification of workers as independent contractors instead of waged or salaried employees—which denies workers benefits and labor protections—is a longstanding arena of conflict (26), and has gotten more controversial in the age of online gig work (i.e., work arrangements mediated by app-based and online platforms) (27, 28).

### Direct vs. Indirect

Direct employment relationships involve two parties: a worker providing labor and an organization purchasing labor. However, economic work arrangements can also involve third parties, in which directive control over work and legal responsibilities may be shared across two (or more) distinct organizations. Perhaps the most common example of indirect (or “triangular”) work arrangements involving third-party employers is the staffing agency model, previously noted, where a worker is hired and employed by a staffing agency. The staffing agency [i.e., the “employing firm,” using Boden et al.'s (16) terminology], in turn, provides the worker for a specific assignment with a host organization (i.e., the “site firm”) that is under contract with the staffing agency. Indirect arrangements can also involve a fourth party, a “lead firm,” which is not onsite but exercises some degree of authority over work processes at the worksite. Examples of business arrangements in which the lead and site firms are distinct entities include franchised and supply chain relationships [see (16) for further discussion]. Indirect arrangements are inherently more complex than direct arrangements, and it is often the case that the entity that controls the work and working conditions is separate from the entity with the clearest legal obligations for protecting workers' health and safety (16, 29). Like misclassification of employees as contractors, organizations can use indirect work arrangements to externalize administrative responsibility and legal liability for a worker, for example, to staffing agencies or vendors rather than their own workers.

These three dimensions serve to distinguish different work arrangements (Table 1), and are important determinants of a worker's experience on the job. Importantly, they are not mutually exclusive and frequently occur simultaneously in work arrangements. For instance, staffing agency jobs are both temporary and indirect since they are of fixed duration, and directive control over work assignments and duration of employment is exercised by the agency's client that is hosting the worker. In comparison, independent contracting jobs, including gig work, are temporary and contracted arrangements but involve direct worker contracts with the client organizations. Subcontracting arrangements, which are similar to staffing agency jobs in that they are temporary and indirect, are further distinguishable. They are also a contracted relationship between the worker and the lead and/or site firm who does not direct their work beyond a contract negotiated in advance. Professional employer organizations, a third-party that hires a client company's employees to become the employer of record, offers another potential configuration (i.e., permanent employees in an indirect relationship). This complexity and the shifting work environments inherent in non-standard arrangements creates challenges in identifying health and safety risks, especially in comparison to permanent and directly overseen employees. Our framework simplifies this complexity with the goal of clarifying connections between work arrangement and health.

**Table 1:** Application of the key features to classify several common work arrangements.

	**Employee**		**Contractor**	
	**Direct**	**Indirect**	**Direct**	**Indirect**
**Permanent**	Standard Employment	Professional employer organization	[Misclassified employees]	–
**Temporary**	Direct hire temp	Staffing agency work	Independent contractor/Gig work	Subcontractor

## Mechanisms of Differential Risk Between Work Arrangements

The complexity of non-standard work arrangements makes interpreting health evidence a challenge. However, the takeaway for OH&S professions is that work arrangements have implications for worker health and safety by potentially intensifying existing hazards or creating new ones within the workplace (9, 22, 29, 30). We suggest five primary mechanisms by which workers in temporary, contractor, and/or indirect work arrangements may experience adverse health outcomes: (1) low levels of social support and provision of resources; (2) lack of familiarity with hazards and equipment; (3) high hazard job placement; (4) reluctance to refuse work; and (5) shifting of responsibility for health and safety from the employer to the worker or client organization.

### Low Levels of Social Support and Provision of Resources

Workers in various forms of non-standard arrangements might be treated differently by employers, clients, or co-workers who may feel “less invested” in them due to the temporary or indirect nature of their jobs. This could diminish or exclude non-standard workers from the information, physical and emotional resources, and social support necessary for working safely (31, 32). For instance, non-standard workers, especially those in temporary positions, may receive less or worse quality safety resources such as training or personal protective equipment (9), as well as face limited opportunities to develop skills or advance their career (33). Furthermore, these workers may find it difficult to integrate into workplace social networks in which site-specific safety information is often communicated (16, 29).

### Lack of Familiarity With Hazards and Equipment

The temporary and indirect nature of non-standard arrangements also contributes to a high probability of frequently changing work settings and job duties. This makes it more difficult for these workers to maintain adequate levels of knowledge of worksite-specific hazard information and safe work practices. Evidence suggests that job tenure, work experience, and access to information about hazards reduces the likelihood of injury (34). Workers in temporary arrangements tend to be younger and have less experience than their permanent counterparts, which may help explain higher injury rates in workers employed by temporary staffing agencies (6, 9, 35).

### High Hazard Job Placement

Organizations may be incentivized to outsource high hazard work to workers in non-standard arrangements, such as contractors or workers employed by staffing agencies, to reduced or eliminate financial liability for potential injury and illness (18, 36). Indeed, evidence suggests that temporary work is, on average, more hazardous (16) and that traditionally high risk industries, such as manufacturing, construction, and agriculture, are increasingly dependent on non-standard workers to fulfill labor needs (6). While some workers placed in high hazard work can manage added risk, like some independent contractors hired for their specialized skills and equipment, other temporary, contracted, or indirectly hired workers may be less able to do so. Additionally, non-employees are less likely to report safety hazards and injuries (36, 37).

### Reluctance to Refuse Dangerous Work

Workers in temporary and contracted work arrangements may feel pressure to perform hazardous tasks or be reluctant to refuse dangerous work out of fear of retribution, lost compensation, or limited opportunities for future employment (1, 29). This may be especially relevant to temporary arrangements, which increase workers' experience of job insecurity (38, 39). Job insecurity is robustly associated with ill physical and mental health (40) and can facilitate worker vulnerability, making it difficult for workers to exercise their formally granted rights (22, 41). For example, the desire to maintain favor with employers could encourage workers to take increased risks to complete work, and discourages reporting of injury or illness by workers (42). Also, workers in non-standard arrangements who lack benefits such as paid sick leave may feel unable to take time off or feel pressure from employers to continue working (15). Evidence suggests that working while ill (i.e., sickness presenteeism) or working long hours without pay out of fear of reprisal (i.e., long hour presenteeism) has health consequences, including increased risk of injury and exposing co-workers, and potentially customers, to infection (43). Furthermore, as labor representation from unions and the ability to collectively organize declines for workers overall, so have health and safety efforts. Fewer workers have accessible avenues for raising concerns in environments where they may risk retaliation (16).

### Diffusion of Responsibility for the Health and Safety of the Worker

In standard employment relationships, ensuring a safe workplace and meeting health and safety regulatory requirements is the clear responsibility of the employer. However, responsibility for safety may become muddled for workers in indirect and contracted arrangements. Shared or confused responsibility over who is required, for example, to provide training and personal protective equipment or report work-related incidents leaves workers vulnerable (1). Moreover, workers suffer when safety measures are not provided, and inadequacies in record-keeping hinder the workers' compensation process (16). A common form of diffusion of responsibility comes when employers misclassify workers as contractors to avoid responsibilities inherent in the employer-employee relationship under employment law, including wage and hour laws and safety protections (1, 16).

## Discussion

The character of work arrangements is an important and understudied determinant of worker health and safety experience on the job. A lack of conceptual clarity has impeded accumulation of knowledge on this topic, and workplace health and safety regulations have not kept pace with labor market trends in new forms of work. To help move research, policy, and practice forward, we highlight three distinguishing features of non-standard work arrangements. We also consider mechanisms by which temporary, contracted, and indirect work arrangements expose workers to harmful workplace conditions.

It is important to note that the three distinguishing features are not the only aspects of work arrangements which influence a worker's health and safety experience. Work schedules, like part-time and shift work, and compensation structures, such as piece work, are also associated with a variety of health outcomes. For example, part-time hours may limit income, benefits (e.g., insurance), and economic security; nighttime shift work is associated with poorer health after a workplace injury than workers who work regular hours (44); and piece work, where workers are paid by the quantity produced, encourages fast-paced work and is associated with poorer psychosocial working conditions, poorer general health, and more pain (45). However, such factors are not uniquely characteristic of non-standard work arrangements—they can apply to any arrangement. They also affect health through mechanisms independent of those described herein. These factors are therefore omitted from our taxonomy, and, instead, we focus primarily on the contractual aspects of work arrangements which have been studied much less thoroughly. Given the complexity and multifaceted nature of relations between workers and employers/clients, more nuanced concepts like precarious employment (22, 46) and employment quality (47), which attempt to characterize more comprehensively the many dimensions of employment relationships, are gaining traction within occupational health. Here, we attempt to make progress by emphasizing three aspects of work arrangements easily identifiable by researchers and practitioners.

We should also note that working conditions vary dramatically by worker, workplace, occupation, and industry, and that non-standard arrangements may benefit some workers. For instance, some workers in non-standard arrangements may experience high control and flexibility in their scheduling or choice of client and have access to premium pay for specialized skills. However, evidence suggests that the growing prevalence in non-standard arrangements is primarily due to employers' preferences, especially cost savings (48, 49). For less skilled workers, non-standard arrangements often mean higher insecurity and worse working conditions. Each of the three features ultimately serve to reduce an organization's obligations to workers conducting labor on their behalf by putting time limits on working relationships (temporary vs. permanent) and externalizing administrative responsibility and legal liability for a worker—either to a third-party (indirect vs. direct) or to the worker themselves (contract vs. employee) (16, 18). Critically, specific worker groups, like women, people of color, immigrants, younger workers, lower-skilled, and lower-educated workers, are disproportionately represented in non-standard work. Workers' individual circumstances can also modify the relationship between work arrangements and health. For example, workers from marginalized populations (e.g., immigrants) may already be at a disadvantage in terms of access to safety information (e.g., via language barriers) or be more reluctant to refuse work for fear of job loss exacerbating the effects of non-standard arrangements. Social distribution of work arrangements, therefore, has important implications for population health disparities (1, 22, 42). We also do not address informal work arrangements, though such work likely has important health implications for workers and may have some overlap with formal non-standard work. The informal sector is challenging to study and falls outside our framework which relies on formal contracts to explain exposure. However, more research is needed to understand the intersections between non-standard work, socio-demographics, and further explore connections with the informal sector (50).

Our framework offers a common language and informs intervention points where OH&S researchers and professionals can conduct research, improve surveillance, and develop programs and policies to protect workers in non-standard work arrangements. Using our simple taxonomy and proposed mechanisms researchers can examine distinct contractual dimensions of work arrangements to advance research on the health implications of non-standard work. For example, perhaps the consistent finding of increased injury risk among temporary staffing agency workers [e.g., (6)] and more mixed results among temporary direct hires [e.g., (51)] can be explained by the indirect nature of the staffing agency model. We can hypothesize that a diffusion of responsibility for worker safety between the multiple involved employers mediates health outcomes for temporary staffing agency workers in ways that may not be applicable to temporary direct hires. To test this hypothesis, researchers can use the three features to develop research questions characterizing arrangement type and identify pertinent mechanisms for increased risk. Additionally, while health research on non-standard work typically focuses on acute work-related health outcomes, these arrangements may also be associated with chronic disease—especially from psychosocial stress (e.g., job insecurity) or increased exposure to unfamiliar hazards. Research on job insecurity frequently explores connections between work arrangements and chronic disease; however, the rise in non-standard work is likely to produce some challenges for work-related chronic disease epidemiology. For instance, occupational histories may be more spotted with short tenures, high numbers of jobsites, and poor documentation of exposures and illnesses (e.g., from third-party employers). Despite these challenges, our framework can assist in exposure assessment to push this line of inquiry forward.

From a practice and policy perspective, OH&S professionals can use this work to identify and support workers in non-standard work arrangements. Workplaces can properly prepare and support non-standard workers for their work by ensuring necessary health and safety resources, screening for qualified skills, facilitating adequate training, and encouraging reporting in the event of an incident. Policy measures can address reluctance to refuse work and improve working conditions by empowering workers through enhanced retaliation laws and encouraging collective organization. Policies can also address the diffusion of responsibility by making host employers financially and legally liable for worker health and safety in indirect arrangements and preventing the misclassification of independent contractors (52). Through considering work arrangement an occupational exposure, OH&S researchers and professionals can leverage OH&S principles to keep pace in addressing health and safety risks in the modern workplace.

## Author Contributions

All authors participated in the conception of the article. AO'C and TP drafted the first version of the article. All authors helped in writing and critically reviewing the article and approved the final draft. The authors agree to be accountable for all aspects of the work's accuracy and integrity. The opinions and conclusions are those of the authors and not necessarily those of the funders or the authors' affiliations.

## Conflict of Interest

The authors declare that the research was conducted in the absence of any commercial or financial relationships that could be construed as a potential conflict of interest.
